# History of a Case of Hernia Cerebri, Consequent on Fracture of the Skull

**Published:** 1821-03

**Authors:** C. Turner Thackrah

**Affiliations:** Member of the Royal College of Surgeons in London; Licentiate of the Society of Apothecaries, &c.


*History of a Case of Hernia Cerebri, consequent on Fracture of the Skull.* By C. Turner Thackrah, Member of the Royal College of Surgeons in London; Licentiate of the Society of Apothecaries, &c.

IN the evening of the 24th of February, 1818, George Jackson, a healthy lad of seven years, had his skull fractured by a blow from a horse's heel.

A portion of cranium, about the size of an egg, situated near the junction of the frontal and temporal bones on the left side, was found to be deeply depressed, and part of it driven edgewise into the cerebrum. The brain was consequently much lacerated, and detached masses of it were scattered on the hair. No considerable hemorrhage had ensued. His pulse was 58, and free from intermission. No vomiting had occurred; the breathing was not stertorous; nor were the pupils wholly devoid of motion. The lad was disposed to continual dosing; but, when roused, his answers were coherent, and his mind apparently rational. He complained, however, of pain in the head.

The fractured portions of bone were successively raised with the forceps and elevator, and, being detached from the dura mater with a scalpel, were readily extracted. Considerable bleeding took place during the operation, but soon subsided. Pulse 74; pain in the head much relieved; the pupils contracted naturally.

The next morning, after passing on the whole a favourable night, he was composed and easy. Pulse 140. I found the wound filled with a mass, apparently composed of brain, membrane, and blood. This, as well as the surrounding integuments, was tender on pressure. The exposed part pulsated strongly. No vomiting had occurred; no intolerance of light; little thirst; and no disposition to delirium. He was bled, and freely purged.

*Vesp.*—The appearance of the wound was not altered, and his general state the same. He had occasionally pain in the head; which, however, he found relieved by bleeding. He vomited immediately after the venesection, and rejected also some doses of his medicine.

*Feb.* 26.—No change in the general symptoms, nor urgency in any. As, however, there was some pain in the head, the application of leeches was ordered. The lad was generally disposed to dose. As the exposed cerebrum had assumed a fungous appearance, lint and simple dressings were applied.

27.—In much the same state as yesterday. Some compression was made on the protuberant brain, by lint and strips of plaster.

*March* 1.—The pressure had not been particularly painful. On the removal of the dressings, the lint usually tore away a portion of the fungoid mass. This occasioned no severe pain; nor did the tumor ever manifest very acute sensibility. It had not increased in bulk. The constitution, however, began to suffer some perturbation. The eyes, of late, had become more susceptible of stimulus; the tongue was white; and the face frequently flushed. Pulse 110—125. He took an occasional purgative.

5.—For the last few days he remained in much the same state; but, indisposition having prevented my seeing him, I found, to my surprise, that the cerebral substance was considerably higher.

7.—The fungus was still on the increase; had attained the size of a large orange; and on its surface were many fissures, from which fetid matter was freely effused. The scalp surrounding the tumor always evinced fully as much tenderness as the substance itself. The lad now complained more of headach, and was more restless. His eyes were generally humid, and slightly inflamed, but the pupil had a healthy contraction. Pulse 130. He had vomited once or twice, but this was traced to error in diet. He felt considerable relief from a refrigerant lotion, with which the head was constantly moistened.

10.—From the fissures and the fetid discharge, I had hoped that a natural separation might have taken place; but, no such change occurring, and the tumor rather increasing in size, I was desirous of removing it with the scalpel. This, however, was disapproved of by my friend Mr. Battye, (who had the kindness to see the patient along with me,) under the apprehension that a profuse and fatal hemorrhage might ensue. He recommended a trial of the sulphate of zinc as an escharotic. Cloths moistened with a solution of this salt were, therefore, freely applied.

11.—The boy remained in the same state; nor had he suffered much pain from the lotion. A portion of the fungus being torn off by the dressings, the tumor manifested a disposition to hemorrhage.

13.—No alteration in the appearance of the fungus; but the soreness and inflammation of the surrounding integuments had extended over the left cheek.

16.—As the escharotic seemed to produce no diminution of the protuberance, mild dressings and a saturnine lotion were substituted. The discharge was copious and fetid. The lad was now allowed to sit up occasionally. He was generally lively, ate moderately, and had no great degree of feverishness.

18.—In the morning I was hastily informed that the boy was in a fit. On my visiting him, however, it appeared that the attack had been one of deliquium, on his rising in bed to take his breakfast.

20.—The fungus was still in some degree on the increase, but became proportionally narrower at its base.

22.—As it had assumed a form sufficiently pendulous to admit a ligature, this mean was adopted. A narrow piece of waxed tape was insinuated round its root, and a moderate compression made. The boy once or twice cried out from violent *abdominal* pain. In a short time, however, he felt no particular uneasiness either in his head or belly. The pulse for a while was somewhat quickened. During the afternoon he suffered some attacks of head-ach, but perfectly retained his senses, and even slept well at night.

25.—As no marked change had taken place either in the constitutional or local symptoms, a second ligature was applied tighter than the first. No particular pain or uneasiness ensued.

28.—The ligature was drawn closer. An abscess was found on the outer edge of the fungus, which discharged about ℥ss. of laudable pus.

*April* 1.—The ligature was again tightened. Severe, though temporary, pain in the head, was the immediate effect. The lad had not borne any but a recumbent posture for the last ten days.

3.—The constriction was again increased. The fungoid mass was now evidently shrinking, laid pendulous on the eye, and was partially covered with a dark crust.

4.—The ligature was again tightened. A very slight hemorrhage succeeded this, as well as the former constrictions. An appearance of cerebral fungus presented itself beneath the ligature. The patient's general health was good.

6.—The neck of the tumor had become narrow. The ligature was drawn closer.

8.—The decayed and shrivelled fungus* was wholly removed; but a fresh one appeared to be rising at its base. To this I applied several compresses of lint, bound down with adhesive strips. The purulent discharge had greatly diminished; nor was there any appearance of abscess.

9.—No considerable pain produced by the compression. The pulsation of the exposed cerebrum was strong.

11.—The fungus began to decrease.

13.—It was considerably lessened, and the granulations on the integuments fast approximated. From this period, the lad was dressed every second or third day, the compression being gradually diminished. The wound at first closed rapidly, but a small ulcer, about the size of a pea, in the middle, was tedious in healing. The boy was quite healthy, and wholly free from pain.

*July* 3.—After passing a very restless night, he had a convulsive attack, which continued for some minutes. On my seeing him in the forenoon, I found him seriously ill, though complaining of little pain. The pulse was 100, intermitting, and irregular. The pupils contracted, not naturally, on stimulus; and the mind was disposed to delirium. Leeches, blisters, and free purgation, were soon, however, productive of decided relief; and in two days every trace of disease was removed.

Since this period the boy has remained in perfect health.

Of this disease, few cases of a favourable result have been recorded; nor has the nature of the tumor, and the means best calculated for its removal, been hitherto ascertained.

In the preceding details, some particulars seem worthy of regard.

1. The fungus was highly vascular, and appeared completely organized.

2. It was connected with abscesses, probably situated near its base.

3. Compression at first seemed to repress its growth.

4. Nature was not able to cast off the full-formed tumor.

5. The application of sulphat of zinc also proved inefficient.

6. Strangulation by a ligature, *gradually tightened*, finally removed the hernia, without inducing any alarming constitutional irritation.

---

* This substance appeared to consist of cerebrum preternaturally vascular. Except in its dark grey hue, it resembled the figure which accompanied Mr. Stanley's paper in the eighth volume of the *Medico-Chir. Trans.*

7. Pressure prevented the growth of the incipient tumor which arose on the destruction of the former.

Hernia cerebri generally induces a fatal event too rapidly to allow the means used in the preceding case. When the constitution becomes seriously affected, excision affords the only prospect of cure. But, should no alarming symptom occur, the surgeon, I conceive, will not err by waiting till the tumor assumes a form suitable for the application of a ligature. The mode in which this remedy should be used, I conclude, from the case related, to be that of gradually increased compression. Had the constriction been severe at first, dangerous affection of the brain would probably have ensued: and I have been recently informed that, in a case occurring in the neighbourhood, such symptoms succeeded on the application of a firm ligature as indicated the propriety of its speedy removal.

*Leeds; December* 1820.

